# A Novel Recombinant Virus-Like Particles Displaying B and T Cell Epitopes of Japanese Encephalitis Virus Offers Protective Immunity in Mice and Guinea Pigs

**DOI:** 10.3390/vaccines9090980

**Published:** 2021-09-02

**Authors:** Muhammad Naveed Anwar, Chunying Jiang, Di Di, Junjie Zhang, Shuang Guo, Xin Wang, Muddassar Hameed, Abdul Wahaab, Donghua Shao, Zongjie Li, Ke Liu, Beibei Li, Yafeng Qiu, Zhiyong Ma, Jianchao Wei

**Affiliations:** Shanghai Veterinary Research Institute, Chinese Academy of Agricultural Sciences, Shanghai 200241, China; dr.naveed903@gmail.com (M.N.A.); xiaoyuanzi17@163.com (C.J.); di.di@merck.com (D.D.); 17317271403@163.com (J.Z.); guo1412634651@outlook.com (S.G.); wang1655609668@outlook.com (X.W.); mudasar386@gmail.com (M.H.); abdul.wahaab@uaf.edu.pk (A.W.); shaodonghua@shvri.ac.cn (D.S.); lizongjie@shvri.ac.cn (Z.L.); liuke@shvri.ac.cn (K.L.); lbb@shvri.ac.cn (B.L.); yafengq@shvri.ac.cn (Y.Q.)

**Keywords:** Japanese encephalitis virus, porcine parvovirus, virus-like particle, immunogenicity

## Abstract

Virus-like particles (VLPs) are non-replicative vectors for the delivery of heterologous epitopes and are considered one of the most potent inducers of cellular and humoral immune responses in mice and guinea pigs. In the present study, VLP-JEVe was constructed by the insertion of six Japanese encephalitis virus (JEV) envelope protein epitopes into different surface loop regions of PPV VP2 by the substitution of specific amino acid sequences without altering the assembly of the virus; subsequently, the protective efficacy of this VLP-JEVe was evaluated against JEV challenge in mice and guinea pigs. Mice immunized with the VLP-JEVe antigen developed high titers of neutralizing antibodies and 100% protection against lethal JEV challenge. The neutralizing and hemagglutination inhibition (HI) antibody responses were also induced in guinea pigs vaccinated with VLP-JEVe. In addition, immunization with VLP-JEVe in mice induced effective neutralizing antibodies and protective immunity against PPV (porcine parvovirus) challenge in guinea pigs. These studies suggest that VLP-JEVe produced as described here could be a potential candidate for vaccine development.

## 1. Introduction

Japanese encephalitis (JE) is an acute infectious disease of the nervous system and is caused by Japanese encephalitis virus (JEV). JEV is the leading cause of encephalitis in Asia pacific regions, and its incidence is expected to increase in some of the world’s most populous countries, such as China, India, Pakistan, Indonesia, and Bangladesh [1,2]. JEV is a zoonotic disease, and similarly to other arboviruses, mosquitoes serve as vectors for the transmission of JEV, whereas pigs and water birds act as amplifying/reservoir hosts for JEV [3]. JEV is a member of the genus Flavivirus in the family Flaviviridae and has a single-strand, positive-sense RNA genome that is nearly 11 kb in length [4]. The World Health Organization has reported that approximately 50,000 JE cases occur each year in South Asia and Western Pacific regions, resulting in more than 10,000 deaths and 15,000 cases of neurological or psychiatric sequelae [5,6].

JEV infection usually occurs as asymptomatic in adult pigs, but there is an increased risk of JE disease in juvenile animals, leading to abortion and stillbirth in pregnant sows [7]. Currently, JEV vaccines used in the swine industry are cell-culture-derived inactivated vaccines, live attenuated vaccines, and chimeric vaccines, all of which are derived from the human live-attenuated SA_14_-14-2 vaccine [8]. These vaccines have effectively decreased JEV morbidity; however, their success has been hampered by high production costs, poor availability, poor long-term immunity, and undesirable side effects [9]. Several studies have suggested that the SA_14_-14-2 vaccine can temporarily give protection for GI JEV infection, but long-term vaccine efficacy might be reduced in GI JEV epidemics or in endemic areas [10,11]. Although JEV immunization has considerably reduced the annual JE burden in many Asian countries [12] as well as minimized the rates of stillbirth and abortion in the swine industry [13], vaccination has only applied to breed boars and sows to prevent abortion and orchitis rather than to block viral circulation, and a high seroconversion in unvaccinated animals is constantly detected from swine farms [14].

VLP is the structure of a virus protein assembly into a hollow particle, and it has the similar natural stability and immunogenicity to the virus, which carries the potential of foreign proteins or peptides. It does not contain virus nucleic acid and is not infectious, which makes it a good indicator of multivalent vaccines [15,16,17]. Immune boosting with VLP vaccines comprising multiple immunogenic epitopes has been shown to overcome JEV disease. The VLP vaccines can be delivered easily and can stimulate effective B cell, T cell, and cytotoxic immune responses, while avoiding potentially hazardous side effects [16].

JEV is a small spherical virion of approximately 50 nm in diameter, which is wrapped in a nucleocapsid and surrounded by an envelope glycoprotein [4]. The JEV coding region encodes a polyprotein precursor which is cleaved by viral and/or host cellular proteases into ten discrete products: three structural proteins (capsid (C), precursor membrane (prM), and envelope (E)) and seven non-structural proteins (NS1, NS2A, NS2B, NS3, NS4A, NS4B, and NS5) [18]. The JEV envelope (E) protein is an exposed structural protein and has multiple immunogenic epitopes, because it has both B cell and T cell epitopes [19,20]. Preferably, an epitope-based vaccine should have both B cell epitopes and T cell epitopes; these are necessary to induce a protective antibody response.

Various platforms have been used for the construction of VLP vaccines. In the present study, we developed a VLP-JEVe vaccine by inserting six JEV E protein epitopes into different loop regions of porcine parvovirus (PPV) VP2 protein. PPV, a member of the *Parvoviridae* family, is the most common cause of reproductive failure in pregnant sows [21,22] and has a single-stranded and negative-sense genome that is composed of three structural proteins: VP1, VP2, and VP3, whereas the capsid is formed mainly by the VP2 protein, which has a neutralizing role for antibody target proteins [23]. Therefore, in the current study, we selected four B-cell-neutralizing epitopes (150–156)-(307–316)-(327–333)-(386–399), together with two T cell epitopes (60–68)-(436–445), based on published epitopes derived from JEV E protein and inserted into PPV VP2 protein by substitutions of their specific amino acids sequences without altering the assembly of the virus. The protective efficacy of this VLP-JEVe vaccine against JEV and PPV challenge was measured in mice and guinea pigs.

## 2. Materials and Methods

### 2.1. Ethics Statement

All animal experiments were carried out according to the Institutional Animal Care and Use Committee of Shanghai Veterinary Research Institute (IACUC No.: Shvri-mo-2019080606). All animal experiments received good humane care, in compliance with animals use according to the animal ethics and the guidelines of China.

### 2.2. Cells, Viruses, Mice, and Guinea Pigs

Baby hamster kidney (BHK-21) cells (CCL-10; ATCC), porcine kidney (PK-15) cells (ATCC CCL-33), and NIH3T3 cells (ATCC CRL-1658) were cultured in Dulbecco’s modified Eagle’s medium (DMEM, Thermo Fisher Scientific, Beijing, China) supplemented with 10% fetal bovine serum (Gibco, Cleveland, TN, USA) at 37 °C in a 5% CO_2_ incubator.

The virulent strain of PPV Nanjing 200,801 (GenBank accession number FJ822038) was propagated and titrated in PK-15 cells.

The virulent strain of JEV N28 (GenBank accession number MH753126) and attenuated JEV vaccine SA_14_-14-2 (GenBank No. AF315119) were propagated and titrated on BHK-21 cells. A 50% lethal dose (LD50) of each JEV strain was tested on three-week-old BALB/c-strain mice by the intraperitoneal inoculation of serially diluted JEV. All the viruses were stored at −80 °C until further use.

Four-week-old female pathogen-free BALB/c mice were purchased from Shanghai SLAC Laboratory Animal Co., Ltd. (Shanghai, China). Female guinea pigs weighing 400–500 g each were housed in groups of three in open boxes on sawdust. The guinea pigs were fed concentrates ad libitum and hay.

### 2.3. Design and Synthesis of Novel VLP Displaying B and T Cell Epitopes of JEV (VLP-JEVe)

In present study, the ideal insertion site for a foreign epitope was selected to efficiently present JEV E protein epitopes on the loop of PPV VP2 protein. There are eight-stranded antiparallel β-barrel motifs which contain many large insertions. These insertions are called loops and have many B cell epitopes. Homology modeling of consensus sequences uses the sequence of one known structure in the protein data banks with other align sequence whose three-dimensional structure is to be predicted. Based on previous studies and homology modeling, we selected a total of six epitopes of JEV E protein (150–156)-(307–316)-(327–333)-(386–399)-(60–68)-(436–445). These epitopes were introduced into different surface loop regions of PPV VP2 by substitutions of specific amino acid sequences. Introduction of a GPGPG spacer between epitopes would have a high likelihood of preventing the formation of most junctional epitopes. The resulting three-dimensional structures of the novel recombinant VLP were analyzed using the molecular graphics software Pymol (Version 1.7.4.4, http://www.pymol.org/ (accessed on 20 August 2021)) [24].

### 2.4. Plasmid Construction

**Figure 1:**
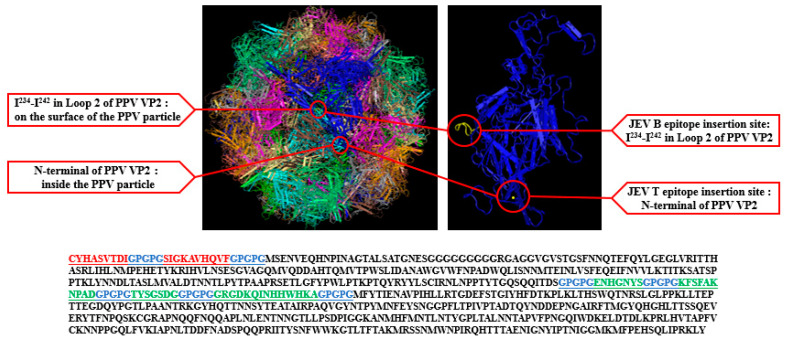
JEV epitopes infusion design and 3D structure of PPV VP2 loop. The N-terminal of PPV VP2 was substituted by the JEV T-epitopes insertion sites (60–68)-(436–445) (red in color), while I^234^-I^242^ in Loop 2 of PPV VP2 was substituted by JEV B epitope insertion sites (150–156)-(307–316)-(327–333)-(386–399) (green in color). GPGPG were used as a spacer between epitopes (blue in color). Porcine Parvovirus Capsid PBD: 1K3V.

First, a genetic codon-optimized VLPe cDNA was synthesized for expression in *Escherichia coli* using the synthetic codon-optimized JEVe (VLP-JEVe) gene created by Sangon, China. This gene was designed for substitutions of amino acid sequences of the N-terminal of PPV VP2 at the JEV T epitope insertion site (60–68)-(436–445), and I^234^-I^242^ in Loop 2 of PPV VP2 was substituted at the JEV B epitope insertion site (150–156)-(307–316)-(327–333)-(386–399), as shown in Figure 1. The fusion gene was PCR-amplified and sub-cloned into the plasmid pCold-I (Takara, Beijing, China). The recombinant plasmid pCold-PPV: VP2-JEVe was transformed into *E. coli* and grown on LB agar and further identified by PCR.

### 2.5. Expression, Purification, and Identification of Recombinant Protein VP2-JEVe

After the recombinant plasmid pCold-PPV:VP2-JEVe was transformed into *E. coli*, the recombinant bacteria was expressed, and the resulting proteins were purified using an Ni affinity system, according to the manufacturer’s protocols (Bio-Rad, Hercules, CA, USA). Purified protein was then analyzed by SDS-PAGE and Western blotting using anti-E (JEV) and anti-VP2 (PPV) polyclonal antibodies (generated in our laboratory), quantified with Bradford protein assay kits (Bio-Rad Laboratories Inc., Hercules, CA, USA), and stored at −80 °C [25].

### 2.6. VLP Assembly

The purified VLP-JEVe (containing B and T cell epitopes) was assembled in vitro and dialyzed in 50 mM of Tris by using NaCl with different concentrations (100 mM, 150 mM, 250 mM, 300 mM) at pH 7.0. VLP-JEVe was loaded onto carbon-coated copper grids and negatively stained with 1% phosphotungstic acid for 10 min [26]. Subsequently, VLPs were examined with a transmission electron microscope (TEM).

### 2.7. Immunization and Challenge in Mice with VLP-JEVe

Four-week-old female BALB/c mice were divided into three groups (13 mice/group). Group 1 was inoculated subcutaneously with VLP-JEVe at a dose of 50 µg at 0.1 mL/mice. The VLP-JEVe was diluted in 50 mL of PBS and emulsified with 50 mL of Complete Freund’s adjuvant. Groups 2 and 3 were inoculated subcutaneously with 1/20 of commercially available live-attenuated SA_14_-14-2 vaccine or PBS at 0.1 mL/mice. Booster immunizations at the same dosage were inoculated at 2-week intervals. The blood samples were taken from the central tail vein on days 0, 7, 14, and 28 of immunization and stored at −20 °C for further experiments. At 28 days post-immunization, spleens were removed aseptically from 3 mice from each sacrificed group for splenocyte culture. Subsequently, 10 mice/group were challenged via an intraperitoneal route with 5 × 10^6^ PFU of lethal JEV N28 strain 28 days after the first immunization, and the survival curves of mice were constructed for the 20 days continuously following challenge. Blood samples from 5 mice/group were taken from the central tail vein 2 and 3 days after challenge to detect viremia levels through qRT-PCR [27].

### 2.8. Immunization and Challenge in Guinea Pigs with VLP-JEVe

Twenty-four female guinea pigs weighing 400–500 g were separated into three groups (8 animal/group). The first group was immunized with 100 mg purified VLP-JEVe (500 μL/guinea pig) mixed with Complete Freund’s adjuvant. The second group was inoculated as a positive control with the inactivated PPV vaccine, whereas the last group was used as a negative control and inoculated with 500 μL of PBS. Tibialis cranialis muscles were used for all inoculations in guinea pigs. Four weeks after the first immunization, the same booster immunization was used. Blood was collected at 0, 14, 28, and 42 days from the forelimb veins of guinea pigs and stored at −20 °C for further experiments. After 42 days from the primary immunization, 3 guinea pigs/group were sacrificed for the proliferation of a spleen lymphocyte assay. The remaining guinea pigs were challenged with a lethal dose of 5 × 10^6^ PFU of PPV Nanjing 200,801 on the 42nd day and monitored for a further 14 days. After this, guinea pigs were sacrificed and the contents of PPV in the spleen were measured through real-time PCR (RT-PCR) [27].

### 2.9. Antibody Testing

To determine the neutralizing antibody titers of JEV from immunized mice, serum was inactivated in a water bath at 56 °C for 30 min, and twofold serial dilutions in RPMI medium were made. These serum dilutions were mixed with an equal ratio of JEV and incubated at 37 °C for one hour, and then subsequently dispensed onto BHK-21 cells grown in 6-well plates. The cells were overlaid with 1.2% methylcellulose (Thermo Fisher Scientific) and incubated at 37 °C for additional 3–5 days. The plaques were stained with 0.5% crystal violet and the titers of neutralizing antibodies were expressed as the serum dilution, yielding a 50% reduction in the plaque number, as described previously [28,29].

Similarly, to determine the ELISA or PRNT_50_ titers of JEV PPV from immunized guinea pigs, serum samples at 28 and 42 days post-immunization were used, as described previously [29,30].

Hemagglutination inhibition (HI) antibody titers were determined as previously described [31]. Briefly, PPV of 25 μL (equivalent to 4 HA units) were mixed with serum in equal ratio and incubated for ten minutes at room temperature. Then, 50 μL of 0.8% mouse erythrocyte suspension was added to the mixture and incubated for a further 30 min; the results describe the highest endpoint dilution producing the complete inhibition of hemagglutination.

### 2.10. Cytokine and Cytotoxic T Lymphocyte Assay

The cytokine profiling assay was carried out as described previously [29]. The extracted supernatants from spleen lymphocytes were used for the study of cytokines (IL-4 and IFN-γ) using ELISA kits (Bogoo, Shanghai, China), as instructed by the supplier.

The cytotoxic T lymphocyte (CTL) assay was performed according to a previous study [31]. Briefly, splenocytes from 3 mice/group were stimulated with 10 μg/mL of VLP-JEVe in RPMI. NIH3T3 cells were used as target cells for the CTL assay and were inoculated with JEV N28 strain 24 h before the assay. After seeding the cells in 96-well plates, the effector-to-target ratio of 50:1 was adjusted, and the supernatant was collected after incubation at 37 °C for 6 h. The activity of lactate dehydrogenase (LDA) was measured through a cytotoxicity assay kit (Promega, Madison, WI, USA).

### 2.11. Spleen Lymphocyte Proliferation Assay

To determine the lymphocyte proliferation assay, 3 guinea pigs/group were used at 28 and 42 days after their immunization. After the separation of spleen lymphocytes using hydroxypropyl methylcellulose (Solarbio, Beijing, China), they were resuspended in RPMI with 10% FBS and stimulated with PPV Nanjing 200,801 strain. Following incubation at 37 °C for 48 h, 10 mL WST-8 per well was added and further incubated at 37 °C for 1 h, while the absorbance of each well was measured at 450 nm. The spleen lymphocyte proliferation assay is normally described as the stimulation index (SI), which is defined as the ratio of the mean values of stimulated to unstimulated wells.

### 2.12. Determination of PPV in Tissue

DNA was extracted in 2 mL of PBS from the spleens of guinea pigs which had been challenged with VLP-JEVe, PPV vaccine, and PBS, using a commercial kit (TIANGEN Biotech, Beijing, China) 42 days after the primary immunization. The DNA content of PPV was measured by qRT-PCR assay with specific primers for the NS1 [27].

### 2.13. Statistical Analyses

Two-tailed Student’s *t* tests and Mann–Whitney U tests were used to determine statistical significance between datasets. Kaplan–Meier survival curves were analyzed by the log-rank test for significance. Probability (*p*) levels less than 0.05 were considered as significant. All statistical calculations were performed using GraphPad Prism software (version 8.0.1, San Diego, CA, USA).

## 3. Results

### 3.1. Expression and Purification Assembly of VLP-JEVe

**Figure 2:**
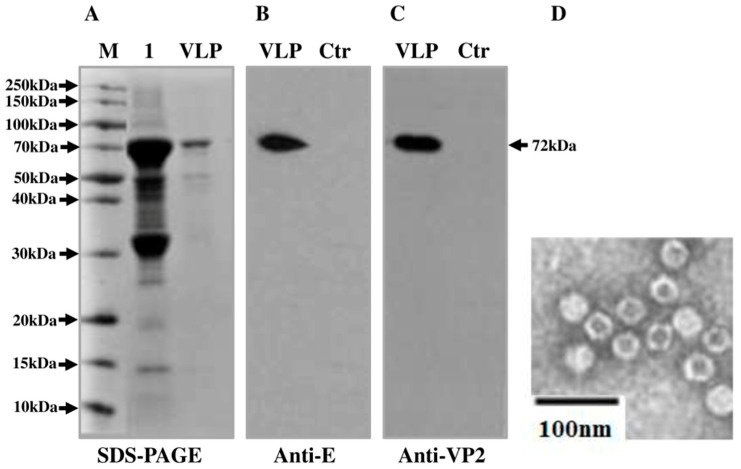
Expression, purification, and characterization of VLP-JEVe. (**A**), SDS-PAGE analysis of the expression of VLP-JEVe in *E.coli*. M: protein marker; lane 1: VLP-JEVe induced with IPTG; lane 2: purified VLP-JEVe. (**B**,**C**), Western-blot analysis of purified VLP-JEVe and control with anti-JEV E and anti-PPV VP2 antibody. (**D**), The purified VLP-JEVe was processed for negative staining and observed with an electron microscope.

The VP2 protein expression in *E. coli* was detected by SDS-PAGE and Western blot analysis. SDS-PAGE analysis revealed the high expression of VLP-JEVe in *E. coli* and gave a band of 72 kDa, which was the expected size of water-soluble His-VP2 protein (Figure 2A). Western blotting indicated that anti-E antibody and anti-VP2 against VP2 protein induced a clear positive reaction with 72 kDa protein (Figure 2B,C). The observed band corresponded to the expected size of the VP2 protein carrying the JEV epitopes, whereas no band was observed in the control group. The Micro BCA™ protein assay gave the total yield of purified VP2 protein as approximately 15 mg for one liter of bacterial culture.

To further analyze the degree of recombinant VLP-JEVe self-assembly into particles, the purified VLP-JEVe was examined by TEM, which showed that purified VLP-JEVe was successfully assembled with a diameter of 25 nm (Figure 2D).

### 3.2. Antibody Response in Mice

**Figure 3:**
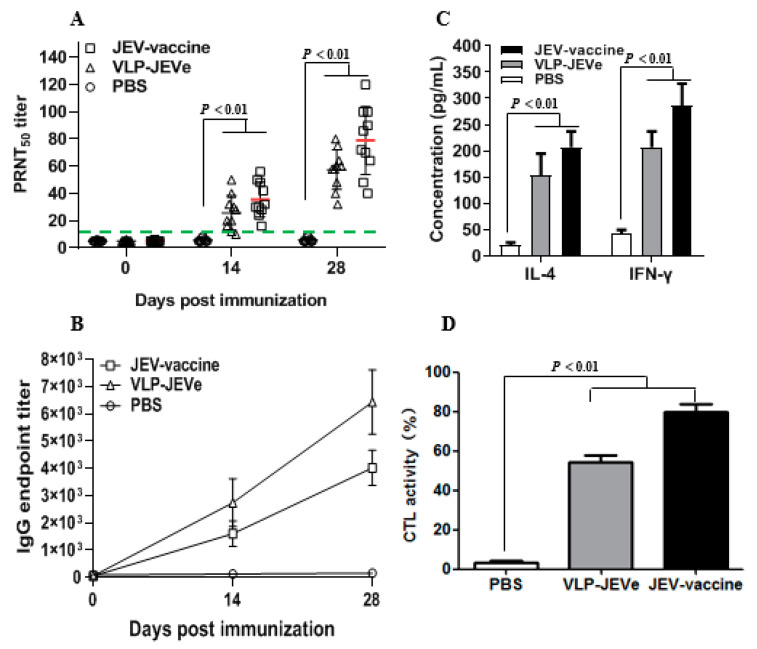
Antibody response in mice. (**A**,**B**), Three groups of mice BALB/c mice were immunized with purified VLP-JEVe, live-attenuated SA_14_-14-2 vaccine and PBS. Serum samples were collected at various time-points, and neutralizing antibody titers or ELISA titers against JEV were determined by PRNT_50_. (**C**), Cytokine detection in splenocyte culture supernatants from vaccinated mice. Lymphocytes isolated from the spleens of vaccinated mice at 42 days were stimulated with VLP-JEVe. After 72 h, the supernatants were collected to detect the concentration of Th2-type cytokine IL-4 and Th1-type cytokine IFN-γ by cytokine ELISA kit. (**D**), CTL activity of splenocyte from vaccinated mice two weeks after the last immunization. Splenocytes from the vaccinated mice were stimulated with VLP-JEVe. After 6 days, the CTL activities were determined using a cytotoxicity assay kit by the measurement of the LDH released. All the experiments were performed in triplicate, with the mean ± standard error (SE) shown. *p* < 0.01 value was compared with groups vaccinated with PBS or VLP-JEVe and SA_14_-14-2 vaccine.

To detect the antibody titers of VLP-JEVe in vitro, the neutralizing antibody assay or ELISA was used. As shown in Figure 3A,B, neutralizing antibodies or ELISA titers were detected at 0, 14, and 28 days post-immunization (dpi), which showed that antibody titers were increased with time. The highest level of neutralizing antibodies was observed in the group inoculated with JEV vaccine SA_14_-14-2, followed by the group inoculated with VLP-JEVe at 14 and 28 dpi, respectively. There was a significant difference between the VLP-JEVe and JEV vaccine groups (*p* < 0.01). No neutralizing antibody activity was exhibited in the PBS control group. These results indicate that VLP-JEVe can induce significant levels of humoral immune responses in mice.

### 3.3. Cytokine and Cytotoxic T Lymphocyte Assays in Immunized Mice

To examine the concentration of cytokines IL-4 and IFN-γ secreted by spleen lymphocytes of immunized mice, the supernatant of cell culture was analyzed by using the cytokine ELISA kit. The variations of each group for IL-4 and IFN-γ are shown in Figure 3B, which were significantly higher for the VLP-JEVe-immunized mice as compared to the PBS group. The cytokine levels in the JEV-immunized group were also observed to be the same as those of the VLP-JEVe-immunized group; however, the contents of IL-4 and IFN-γ differed between groups.

Cytotoxicity assays were performed to determine the CTL level of splenocytes. As shown in Figure 3C, the CTL activities of VLP-JEVe and JEV vaccine groups were significantly higher as compared to the PBS group, with mean titers of 55 ± 4%, 80 ± 5% and 3 ± 3%, respectively.

### 3.4. Protection against JEV Challenge in Mice

**Figure 4:**
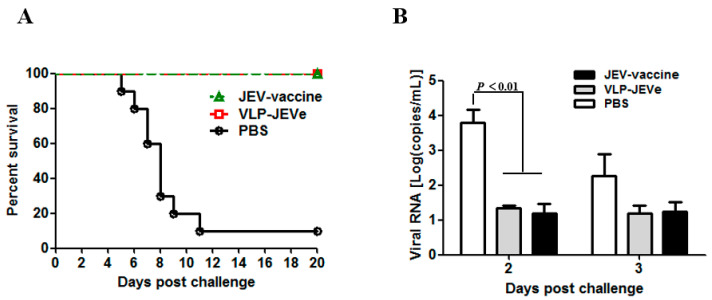
Immunization and challenge in mice. (**A**), The mice were vaccinated with purified VLP-JEVe, live-attenuated SA_14_-14-2 vaccine and PBS and challenged via i.p route with a lethal dose of 5 × 10^6^ PFU of JEV. The mice were monitored daily for 20 days. Kaplan–Meier test was used for survival analysis. (**B**), After challenge of two weeks of immunization of mice, the spleens were isolated and quantitative real-time PCR was performed to measure the virus content. The lower detection limit of the assay is indicated by the dashed line. Data are shown as mean ± SEM, and statistical differences between vaccinated groups and PBS groups were indicated by *p* < 0.01.

To determine the protective efficacy of VLP-JEVe and JEV vaccines, all the immunized mice were inoculated with 5 × 10^6^ PFU of lethal JEV N28 strain through an i.p route. Compared to the PBS group, the mice immunized with VLP-JEVe and JEV vaccines exhibited complete protection (100%) against a virulent challenge of JEV, as shown in the Figure 4A.

After a two-week immunization challenge, the mouse spleens were isolated, and quantitative RT-PCR was performed to measure the virus contents. As shown in Figure 4B, the PBS control group had higher viral loads as compared to the VLP-JEVe and JEV vaccine-immunized groups, indicating that VLP-JEVe and JEV vaccines offered protection against the lethal challenge of JEV. Determination of the detection limit was based on the lowest level at which viral RNA was detected.

### 3.5. Antibody Response in Guinea Pigs

**Figure 5:**
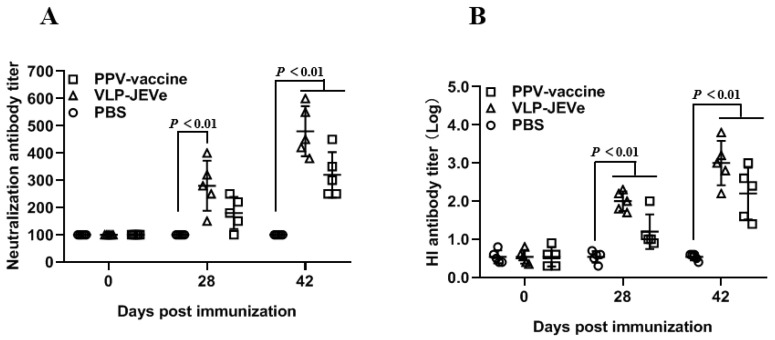
VLP-JEVe specific humoral immune response in guinea pigs. Three groups of guinea pigs were vaccinated with VLP-JEVe, PPV inactivated vaccine, and PBS as control. The blood samples were collected at the indicated time-points for virus neutralizing antibody (**A**) and hemagglutination inhibition assays (**B**). Titers are represented as mean ± SEM and the *p* < 0.01 indicates the statistical difference between vaccinated and PBS groups.

This model is used to test the efficacy against PPV infection, which apparently does not cause pathogenicity in mice. Therefore, to determine the antibody immune response, all the guinea pigs were vaccinated with either VLP-JEVe, inactivated PPV vaccine, or PBS as control, and the neutralizing and HI antibody responses were measured at 0, 28, and 42 days after immunization. The highest levels of neutralization antibody (Figure 5A) and HI antibody titers (Figure 5B) were detected in the VLP-JEVe-immunized group, followed by the PPV vaccinated group, but there was not any response in the PBS control group. These results demonstrated that the highest antibody titers were induced in the VLP-JEVe-vaccinated guinea pigs; the titers gradually increased up to 42 days following the booster immunization.

### 3.6. T Lymphocyte Proliferation and the Dynamic Changes in PPV Contents in the Spleen of Immunized Guinea Pigs

**Figure 6:**
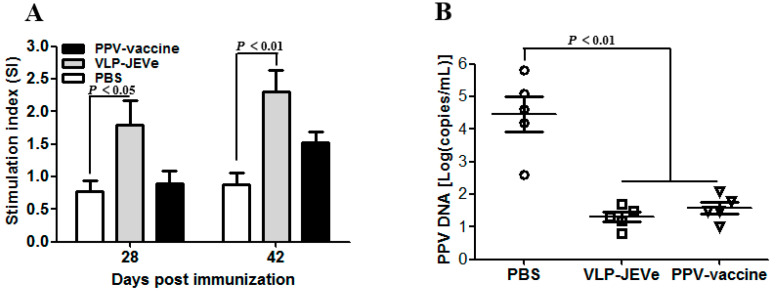
(**A**), Splenocyte proliferation following in vitro stimulation. After immunization with VLP-JEVe, PPV inactivated vaccine, the splenocytes were collected at 28 and 48 days from 3 sacrificed guinea pigs from each group, and then were stimulated with PPV 100 TCID_50_. After 48 h of stimulation, WST-8 was added and the proliferation responses were detected by the stimulated index (SI). (**B**), Protection from PPV virus challenge in guinea pigs. The guinea pigs were challenged with a lethal dose of 5 × 10^6^ PFU of PPV at 42 days after primary immunization and monitored for further 14 days. The DNA was extracted from the spleen samples of guinea pigs and the copy number of PPV genomic DNA was determined by quantitative real-time PCR assay. The lower detection limit of the assay is indicated by the dashed line. Data are shown as mean ± standard error. The statistical differences between vaccinated groups and PBS groups were indicated by *p* < 0.01, *p* < 0.05.

To study the lymphocyte proliferation assay in guinea pigs, extracted lymphocytes 28 and 42 days after their primary immunization were stimulated with PPV Nanjing 200,801 strain. As shown in Figure 6A, the highest level of stimulation index (SI) was observed in the group inoculated with VLP-JEVe, followed by the group inoculated with the inactivated PPV vaccine; however, there were significant differences between the VLP-JEVe and negative control PBS groups.

Virus loads in the tissues of guinea pigs infected with PPV were considered as an important indicator for vaccine-induced serum antiviral effects [31]. Therefore, guinea pigs in the two groups were immunized with VLP-JEVe and inactivated PPV vaccines, all three groups, including the PBS control group, were challenged with PPV Nanjing 200,801 virus, and virus copies in the spleen were quantified using RT-PCR (Figure 6B). The results showed that the PBS group had remarkably higher viral loads than the other groups, indicating that guinea pigs immunized with inactivated VLP-JEVe and PPV vaccines were protected against virulent PPV challenge.

## 4. Discussion

Vaccination is the most effective way to control JEV epidemics in pigs [13,32]. The safety and effectiveness of a JEV live-attenuated vaccine in pregnant sows remains uncertain [33], and formalin inactivation can change the antigenic E protein structure [34]. Despite the fact that current vaccines are reducing the burden of JEV, the virus is still continuously spreading beyond its traditional boundaries [35]. Therefore, a non-infectious vaccine of JEV could be a suitable candidate, especially with VLPs, because they function as an effective antigen but without viral genome or other potentially toxic viral gene products [36]. There are several advantages of using VLPs: they eliminate the need for maintaining virus stocks or virus reservoirs, preventing accidental release of the virus; the maintenance of containment facilities is easier; and, above all, they are easier to handle. VLPs are generally stable vaccines, which are easier to store or transport at diverse temperatures. Additionally, this stability reduces the cost of vaccines by allowing a longer shelf life and reduces energy costs associated with cold-storage [37]. This approach has been successfully used in various infectious diseases, such as human papillomavirus (HPV) [38], hepatitis B virus [39], and Neisseria meningitides infection [40]. Therefore, in this study, a novel VLP vaccine displaying B and T cell epitopes of JEV was proposed as a possible candidate vaccine with a significant capacity to induce protection against a JEV challenge in mice, with some protective immunity against PPV challenge in guinea pigs.

The envelope (E) protein of JEV is associated with virus binding to cellular receptors, membrane fusion, and it has both B and T cell epitopes [41]. Ideally, an epitope-based vaccine should have both B and T cell epitopes; therefore, in our present study, we selected four B-cell-neutralizing epitopes (150–156)-(307–316)-(327–333)-(386–399), together with two T cell epitopes (60–68)-(436–445) from the JEV envelope protein to develop an epitope-based vaccine (VLP-JEVe).

The anti-JEV neutralizing antibodies primarily recognized E protein [42]. In this study, a significant level of humoral immune response was induced from the sera of immunized mice with VLP-JEVe, as well as in mice immunized with inactivated JEV vaccines. Studies have demonstrated that neutralizing antibodies are important for protection against JEV infection [43], and PRNT_50_ titers of ≥1:10 are recognized as protective [29]. The highest level of neutralizing antibodies was observed in the group inoculated with the JEV SA_14_-14-2 vaccine, followed by the group inoculated with VLP-JEVe at 14 and 28 dpi, respectively, which indicates the potential of VLP-JEVe as a vaccine candidate against JEV infection.

Cell-mediated immune responses should always be produced from VLP vaccination [31]. The secretion of IL-4 and IFN-γ indicated the cellular response produced in VLP-vaccinated mice. Although the secretion of IL-4 and IFN-γ for VLP-JEVe was lower than for the JEV SA_14_-14-2 vaccine, the levels of IL-4 and IFN-γ were significantly higher than the PBS group. Virus-specific CTLs are considered critical for the recovery of infection and clearance from JEV [44]; therefore, because the CTL activity in the VLP-JEVe and JEV vaccine groups was higher compared to the PBS group, this indicated a higher degree of protection. Moreover, analysis of the protective efficacy of VLP-JEVe and SA_14_-14-2 indicated a complete protection from challenge by its virulent parental strain. JEV E and prM proteins are known to produce a range of protective immune responses, including JEV-specific B and CTLs in mice and pigs [8]. Taken together, these results suggest that VLP-JEVe can be used as a potential vaccine candidate against JEV infection.

Guinea pigs are a well-established animal model to study the effectiveness of inactivated PPV vaccines, and previous study has revealed that VLPs can provide protection to guinea pigs from its virulent parental PPV challenge if the antibody titer of HI is greater than 1:16 [31]. In this study, the highest levels of PRNT_50_ and HI antibody titers were detected in the VLP-JEVe-immunized group followed by the inactivated PPV-immunized group, although antibodies were not present in the PBS control group. The lymphocyte proliferation response is also a good indicator of recovery from viral infection [31]; as such, the highest level of SI was detected in the VLP-JEVe group, followed by the group inoculated with the inactivated PPV vaccine. PPV mostly causes fetal and early neonatal mortality, and the tissue contents of PPV in infected guinea pigs are a good indication of the antiviral effects of any vaccine [45]. The genomic copy numbers of PPV in VLP-JEVe-inoculated guinea pigs were significantly lower than in the PBS control group, which clearly demonstrated that VLP-JEVe could serve to protect the animals from PPV virulent challenge. According to these data, VLP-JEVe performs better than a PPV vaccine in guinea pigs and perhaps also for other existing PPV vaccines, which needs to be tested in the future [45,46].

## 5. Conclusions

In this study, we developed a novel VLP-JEVe vaccine containing B and T cell epitopes of JEV. The vaccination of mice and guinea pigs with VLP-JEVe induced remarkable humoral and cell-mediated immune responses and provided complete protection against a lethal JEV challenge in mice. The guinea pigs vaccinated with VLP-JEVe exhibited effective neutralizing and hemagglutination inhibition responses when challenged with the PPV strain. In addition, VLP-JEVe significantly reduced the viral load in tissues subsequent to a lethal PPV challenge in guinea pigs. It is positive to see that vaccination produced a neutralizing response against JEV in mice and a neutralizing response against PPV in guinea pigs. Testing guinea pig sera against JEV and mouse sera against PPV could indicate the potential for the vaccine to work across multiple species. Thus, VLP-JEVe may be a potential vaccine candidate for the prevention of JEV and PPV infection. Our data pave the way for the safe, efficacious, and economic development of new VLPs vaccine for JEV and other flaviviruses.
